# Computational Fluid Dynamics Characterization of Two Patient-Specific Systemic-to-Pulmonary Shunts before and after Operation

**DOI:** 10.1155/2019/1502318

**Published:** 2019-02-03

**Authors:** Neichuan Zhang, Haiyun Yuan, Xiangyu Chen, Jiawei Liu, Qifei Jian, Meiping Huang, Kai Zhang

**Affiliations:** ^1^School of Mechanical and Automotive Engineering, South China University of Technology, Guangzhou 510640, Guangdong, China; ^2^Department of Cardiac Surgery, Guangdong Cardiovascular Institute, Guangdong Provincial Key Laboratory of South China Structural Heart Disease, Guangdong Provincial People's Hospital, Guangdong Academy of Medical Sciences, Guangzhou, China; ^3^Department of Catheterization Lab, Guangdong Cardiovascular Institute, Guangdong Provincial Key Laboratory of South China Structural Heart Disease, Guangdong Provincial People's Hospital, Guangdong Academy of Medical Sciences, Guangzhou, China; ^4^Guangdong Cardiovascular Institute, Guangdong Provincial Key Laboratory of South China Structural Heart Disease, Guangdong Provincial People's Hospital, Academy of Medical Sciences, School of Medicine, South China University of Technology, Guangzhou, China

## Abstract

Studying the haemodynamics of the central shunt (CS) and modified Blalock–Taussig shunt (MBTS) benefits the improvement of postoperative recovery for patients with an aorta-pulmonary shunt. Shunt configurations, including CS and MBTS, are virtually reconstructed for infants A and B based on preoperative CT data, and three-dimensional models of A, 11 months after CS, and B, 8 months after MBTS, are reconstructed based on postoperative CT data. A series of parameters including energy loss, wall shear stress, and shunt ratio are computed from simulation to analyse the haemodynamics of CS and MBTS. Our results showed that the shunt ratio of the CS is approximately 30% higher than the MBTS and velocity distribution in the left pulmonary artery (LPA) and right pulmonary artery (RPA) was closer to a natural development in the CS than the MBTS. However, energy loss of the MBTS is lower, and the MBTS can provide more symmetric pulmonary artery (PA) flow than the CS. With the growth of infants A and B, the shunt ratio of infants was decreased, but maximum wall shear stress and the distribution region of high wall shear stress (WSS) were increased, which raises the probability of thrombosis. For infant A, the preoperative abnormal PA structure directly resulted in asymmetric growth of PA after operation, and the LPA/RPA ratio decreased from 0.49 to 0.25. Insufficient reserved length of the MBTS led to traction phenomena with the growth of infant B; on the one hand, it increased the eddy current, and on the other hand, it increased the flow resistance of anastomosis, promoting asymmetric PA flow.

## 1. Introduction

Systemic-to-pulmonary shunts (SPSs), consisting of an artificial shunt connecting the aorta to the pulmonary artery, are palliative operations for neonates with cyanotic congenital heart disease such as Tetralogy of Fallot, pulmonary artery hypoplasia, and hypoplastic left heart syndrome [1, 2]. For patients with diminished pulmonary blood flow, an artificial systemic-to-pulmonary artery shunt has frequently been used to provide blood flow from systemic circulation to pulmonary circulation. MBTS with a shunt connecting the innominate artery (IA) to the right pulmonary artery (RPA) and CS with a shunt connecting the ascending aorta (AAO) to the main pulmonary artery (MPA) are commonly used SPS in clinical settings.

Currently, computer fluid dynamics have been increasingly used to study the postoperative haemodynamics of SPS [3]. They allow for the comparison of the haemodynamic characteristics of different shunt configurations [4] and studying the effects of different anastomosis methods on the haemodynamics of the MBTS [5]. Mathematical modelling is used to predict the postoperative haemodynamic parameters [6]. Based on mathematical models such as the lumped parameters model (LPM), there are many studies covering the effects of the shunt diameter's size and its orientation in SPS haemodynamics [7–9].

This study utilized clinical data from two infants, A and B, born with pulmonary artery stenosis. Infant A presented with CS in the clinic when he was three months old, and CT data were collected before and 11 months after surgery; infant B presented with MBTS in the clinic when he was 12 days old, and CT data were collected before and 8 months after surgery. Notably, we reconstructed shunt configurations including CS and MBTS virtually based on the CT data of preoperative patients to analyse the haemodynamic characteristics of CS and MBTS. Furthermore, we reconstructed three-dimensional postoperative models by postoperative CT data of A and B to compare the haemodynamics of two infants before and after surgery and analyse the effects of CS and MBTS on the postoperative development of the pulmonary artery.

## 2. Materials and Methods

### 2.1. Diameters of Shunts

The decision about shunt size is mostly based on clinical experience through the patient's body weight [10]: 3 mm grafts are used for infants weighing <3 kg and ≥3.5 mm shunts for 3∼6 kg patients. Considering the specific condition of patients, a shunt with a diameter of 3.5 mm was selected for our study.

### 2.2. 3D Reconstruction

**Figure 1:**
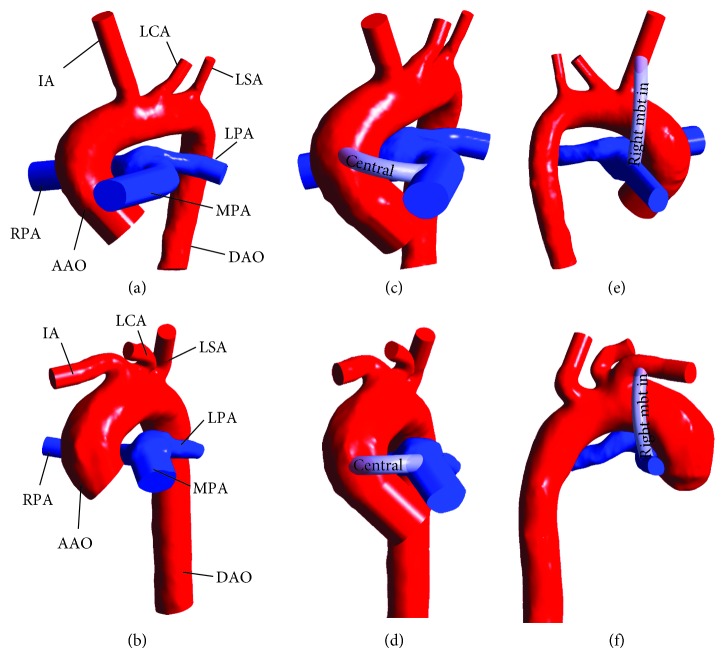
Three-dimensional neonatal arteries model of infants A ((a) model A) and B ((b) model B) and central shunt (CS) configuration established for infants A ((c) A-CS) and B ((d) (B-CS)), modified Blalock–Taussig shunt (MBTS) configuration established for infants A ((e) A-MBTS) and B ((f) B-MBTS). In order to simplify the model, we neglected independent branching vessels. The remaining vessels include ascending aorta (AAO), innominate artery (IA), left carotid artery (LCA), left subclavian artery (LSA), descending aorta (DAO), main pulmonary artery (MPA), left pulmonary artery (LPA), and right pulmonary artery (RPA). The central shunts are placed between AAO and MPA. The right-modified Blalock–Taussig (MBT) innominate shunt connects IA to RPA.

3D anatomical data from a 155 multislice CT of infant A and a 138 multislice CT of infant B were provided by the Guangdong Provincial People's Hospital. As is shown in Figure 1, model A represents three-dimensional neonatal arterial model of infant A, and A-CS (A-MBTS) indicates that CS (MBTS) location configurations was created virtually for model A. Model B represents the three-dimensional neonatal arterial model of infant B, and B-CS (B-MBTS) indicates that CS (MBTS) location configurations were restructured virtually for model B.

### 2.3. Calculation Methods

**Figure 2:**
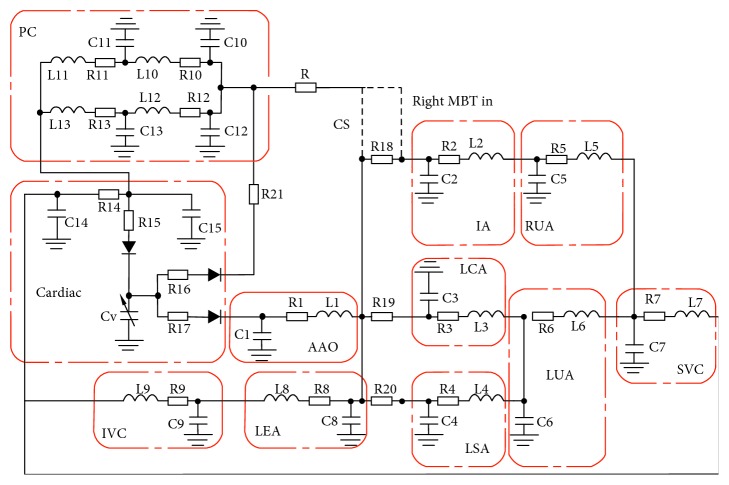
Lumped parameters model. LPM is made up of cardiac and pulmonary circulation (PC), ascending aorta (AAO), innominate artery (IA), left carotid artery (LCA), left subclavian artery (LSA), right upper extremity artery (RUA), left upper extremity artery (LUA), superior vena cava (SVC), lower extremities artery (LEA), and inferior vena cava (IVC).

Several numerical studies indicate that the influence of shear thinning properties of blood is not significant for the flow in large arteries under steady flow conditions [11–13]. Additionally, studies showed that, under steady flow conditions, the Newtonian model is certainly a good approximation in regions of midrange to high shear, with the debate centring on whether the fact that it underestimates WSS in regions of low shear is biologically significant [14]. Non-Newtonian properties of blood were exhibited by blood typically only at shear rates lower than 100 s^−1^ [15]. In this study, regions of interest are large arteries, and the shear rate in the region of interest is greater than 100 s^−1^ for systemic-to-pulmonary shunt [16]. Therefore, the blood was assumed to be an incompressible Newtonian fluid with a density of 1060 kg/m^3^ and viscosity equal to 0.0035 kg/(m·s) [17] with 3D domains being rigid walled [18, 19]. To compute haemodynamic variables for different shunt configurations, LPM was built up based on the relevant studies [5, 20, 21]. Postoperative LPM (Figure 2) can be divided into four parts: cardiac, systemic circulation, and pulmonary circulation and shunt.

The LPM consists of resistors (R), inductors (L), and capacitors (C), which represent the viscous resistance, inertance, and the compliance of the vessel [22], and diodes in the circuit were used to simulate the cardiac valves.

Atria are represented in terms of a constant compliance (C14 and C15) since atrial contractility is discarded [20]. Cardiac pressures are considered to be composed of active and passive parts. In order to represent the rhythmic contraction of cardiac circulation, the relation function between pressure and volume was described as follows:

(1) $$\begin{array}{c} E\left(t\right)=\frac{P_{\text{sv}}\left(t\right)}{V_{\text{sv}}\left(t\right)-V_{0}}, \end{array}$$

where *E*(*t*) [23] meant time-varying elastance function, with unit mmHg/mL. *P*_sv_(*t*) and *V*_sv_(*t*) represents time-varying ventricle pressure and time-varying volume of ventricle, respectively. *V*_0_ was the initial value of ventricular volume. (*t*) can be computed by the following function:

(2) $$\begin{array}{c} E\left(t\right)=\left(E_{\text{max}}-E_{\text{min}}\right)\cdot E_{n}\left(t_{n}\right)+E_{\text{min}}, \end{array}$$

where *E*_max_ and *E*_min_ were related with ventricle pressure and volume in end systole and diastasis, respectively. In this study, *E*_max_ = 2.5118 and *E*_min_ = 0.0458 [5]. These values were kept constant during the subsequent calculations. *E*(*t*_*n*_) (double Hill function [24]) was described as follows:

(3) $$\begin{array}{c} E_{n}\left(t_{n}\right)=1.55\cdot \left[\frac{{\left(t_{n}/0.7\right)}^{1.9}}{1+{\left(t_{n}/0.7\right)}^{1.9}}\right]\cdot \left[\frac{1}{1+{\left(t_{n}/1.17\right)}^{21.9}}\right], \end{array}$$

where *t*_*n*_=*t*/(0.2+0.15  *t*_c_) (*t*_c_ was one cardiac cycle interval, and it was set as 0.5 s according to the specific patient).

The values of the LPN elements that refer to relevant studies [5, 17, 21] are shown in Table 1. Also, Ren and Ding [5, 17] utilized clinical data and proved the rationality of the values of the parameters in LPM.

For physiologic circulation, it is observed that the time-averaged velocity field of pulsatile flow does not show remarkable differences to steady-state results [25]. The boundary condition was set as the average over a cardiac cycle, with boundary conditions of CS and MBTS shown in Tables 2 and 3. In addition, the boundary condition was assumed to be the same for infants A and B enabling comparison of haemodynamics in a different three-dimensional anatomy.

### 2.4. Energy Loss, LPA/RPA Ratio, and Shunt Ratio

The fundamental purpose of a systemic-to-pulmonary artery shunt is to provide the appropriate blood flow from systemic circulation to pulmonary circulation to promote the development of PA. LPA/RPA ratio (*R*_LPA/RPA_) and shunt ratio (*η*) are important parameters to evaluate shunt configurations:

(4) $$\begin{array}{c} R_{\text{LPA}/\text{RPA}}=\frac{Q_{\text{LPA}}}{Q_{\text{RPA}}},\\ \eta =\left(\frac{Q_{\text{Shunt}}}{Q_{\text{AAO}}}\right)\times 100\%, \end{array}$$

where *Q*_LPA_, *Q*_PRA_, *Q*_Shunt_, and *Q*_AAO_ indicate the volumetric flow rate at LPA, RPA, shunt, and AAO.

Energy loss *W*_loss_ is an indicator for evaluating haemodynamic efficiency. The smaller the energy loss, the higher the energy conversion efficiency of shunt configurations [26, 27]:

(5) $$\begin{array}{c} W=Q_{\text{v}}\left(P+\frac{1}{2}\rho v^{2}\right),\\ W_{\text{loss}}=\sum W_{\text{inlet}}-\sum W_{\text{outlet}}, \end{array}$$

where *Q*_v_, *P*, *ρ*, and *v* indicate the volumetric flow rate, static pressure, density, and mean velocity. ∑*W*_inlet_ is the sum of the inlet energy, and ∑*W*_outlet_ is the sum of the outlet energy.

## 3. Result

**Figure 3:**
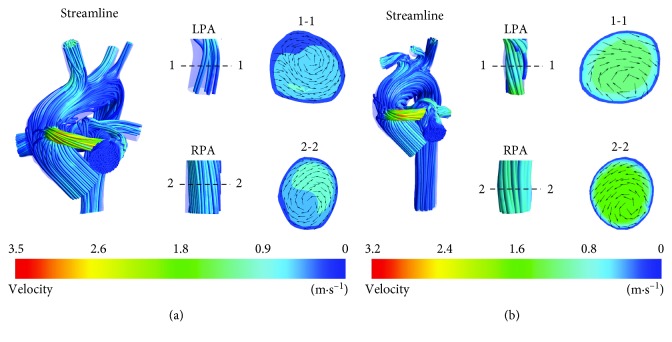
Streamline and velocity vector plots at CS: (a) A-CS; (b) B-CS.

**Figure 4:**
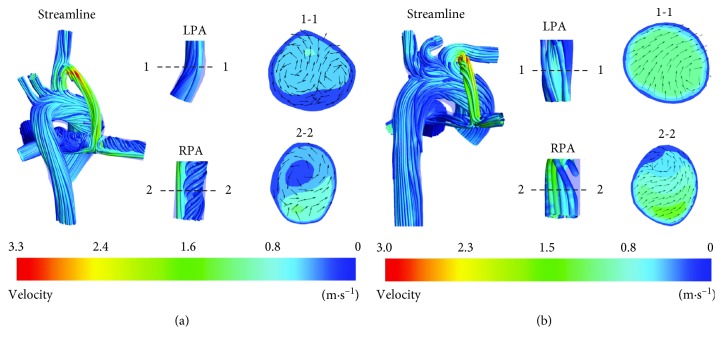
Streamline and velocity vector plots at MBTS: (a) A-MBTS; (b) B-MBTS.

3D blood flow streamlines clearly show the flow state of SPS in Figures 3 and 4. A velocity vector diagram at corresponding sections and partial enlargement of streamline of LPA and RPA are shown to describe the complex flow structures in PA, which is closely related to abnormal growth of PA [4].

In Figure 3, aortic blood with high pressure and flow rate flows through the shunt and mixes with pulmonary blood in MPA. The turbulence intensity in MPA is high, and swirls occurred near anastomosis of MPA. Velocity distribution in LPA and RPA is relatively uniform, which is close to natural development. The blood flow rate of RPA in A-CS was 100% higher than that of LPA, and the blood flow rate of RPA in B-CS was 5% lower than that of LPA, which indicated that the preoperative PA structure has an important influence on symmetrical flow of LPA and RPA for patients with CS. Therefore, the arterial structure of specific patients should be considered when LPA/RPA is an important parameter affecting shunt operation. The shunt ratios of A-CS and B-CS are 34.61% and 34.19%, respectively. The ratio for infants A and B was similar when CS was performed for two infants due to an identical shunt size.

In Figure 4, aortic blood with high pressure and flow rate flows through the shunt and mixes with pulmonary blood in RPA, which leads to high vorticity regions in RPA. Velocity distribution in LPA is relatively uniform, while high vorticity regions in RPA result in an uneven velocity distribution in RPA. Pulmonary blood flow of RPA in A-MBTS was 66% higher than that of LPA, and pulmonary blood flow of RPA in B-MBTS was 1% higher than that of LPA, which demonstrates that the arterial structure also has an important effect on flow distribution of LPA and RPA for MBTS. Even though the length and curvature of shunts are different, the shunt ratio of A-MBTS and B-MBTS are close; the shunt ratios of A-MBTS and B-MBTS were 25.29% and 26.14%, respectively, which indicated that the main factors affecting the shunt ratio of MBTS is the diameter of the shunt. Energy loss of CS was greater than that of MBTS for two infants. For infant A, energy loss of CS and MBTS was 0.16 W and 0.13 W, respectively; for infant B, energy loss of CS and MBTS was 0.11 W and 0.08 W, respectively. It is notable that the flow state of SPS without additional pulmonary blood flow (APBF) [4] is different from the flow state of SPS with APBE. When there is still APBE, energy loss of MBTS is lower than that of CS, while the conclusion is just the opposite when MPA was transected [4]. When CS (MBTS) was performed for patient A, the LPA/RPA ratio was 0.49 (0.59); when CS (MBTS) was performed for patient B, the LPA/RPA ratio was 1.05 (0.99). This shows that the RPA/LPA ratio of MBTS approaches unity when compared with CS; that is, MBTS can provide a more symmetrical flow between LPA and RPA. The shunt ratio of CS is approximately 30% higher than that of MBTS for infants A and B, which indicates that CS has a greater chance of congestive heart failure. There is another point that the diameter of the MBT shunt is limited by the size of the RPA; CS could be preferred for patients with narrow PA to prevent thrombosis due to small size shunts [10].

**Figure 5:**
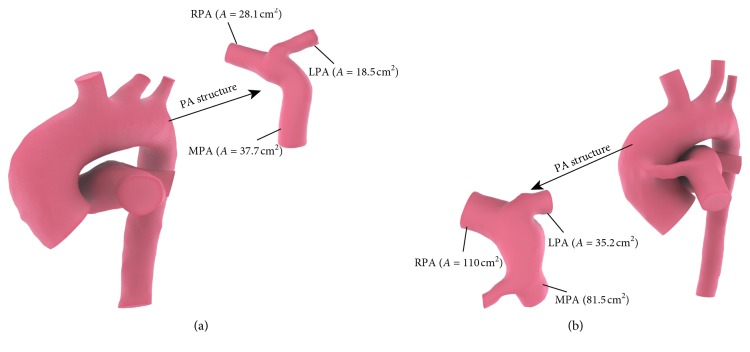
Arterial structure of infant A before (a) and after (b) surgery.

Infant A was presented with CS in the clinic.  Figure 5(a) shows the preoperative arterial geometry of infant A, and Figure 5(b) shows the arterial geometry of patient A 11 months after operation. Blood flow in RPA was 50% higher than blood flow in LPA after the creation of the central shunt virtually in simulation results of A-CS. Prediction based on simulation results indicated that when CS was performed for infant A, RPA tends to develop better than LPA. After 11 months, the cross-sectional area of MPA increased by 70%, and the cross-sectional area of RPA and LPA increased by 290% and 90%, respectively. The results of PA growth after 11 months were that the development of RPA was better than LPA, which is consistent with the prediction based on the simulation results of A-CS.

**Figure 6:**
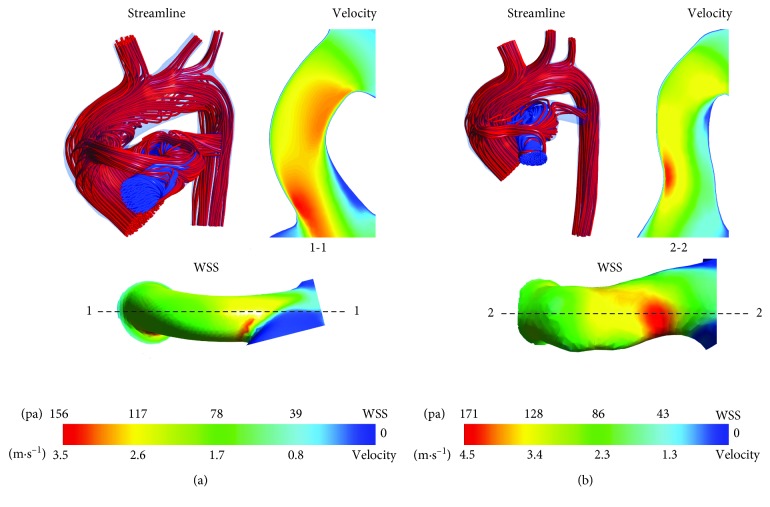
Streamline, velocity contour, and wall shear stress (WSS) plots at CS before (a) and after (b) surgery for infant A.

The boundary condition of CS postsurgery was set as the same in Table 1. On being subjected to high pressure gradients and varying flow pulsatility, SPS often develops uneven intraluminal narrowing or curvature distortion during the first months after implantation [28]. As is shown in Figure 6, maximum velocity in the shunt increases and shunt ratio decreases from 34.61% to 18.17% due to distortion of the shunt. In addition, increase of maximum shear stress and high shear stress region may lead to thrombosis and a series of complications such as intimal hyperplasia [29, 30] after distortion of the shunt. Abnormal preoperative PA structure for CS, such as size differences between LPA and RPA, bending of MPA, and sharp angle between RPA and MPA, will lead to asymmetric LPA/RPA flow. There is a vicious spiral: asymmetric LPA/RPA flow results in asymmetric development of LPA and RPA; in turn, asymmetric development of LPA and RPA will lead to more asymmetric LPA/RPA flow. The blood flow rate of RPA was 100% higher than that of LPA in A-CS. With the growth of the patients, the blood flow rate of RPA was 300% higher than that of LPA after 11 months.

**Figure 7:**
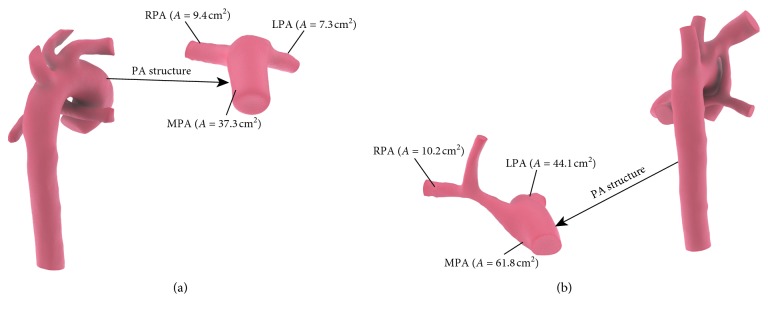
Arterial structure of infant B before (a) and after (b) surgery.

Infant B received an MBTS in the clinic.  Figure 7(a) shows the preoperative arterial geometry of infant B, and Figure 7(b) shows the arterial geometry of patient B 8 months after operation. It can be seen from Figure 7 that, with the growth of infant B, IA gradually grows upward and traction occurred in RPA, which resulted in the bending of the RPA. Preoperative B-MBTS simulation results showed that there is a symmetric pulmonary artery flow after MBTS was performed for infant B, and therefore, LPA and RPA can develop symmetrically after operation. However, the cross-sectional area of MPA increased by 60%, and LPA and RPA increased by 500% and 30% after 8 months. The development of LPA was being obviously better than that of RPA, which is inconsistent with the simulation results of B-MBTS.

**Figure 8:**
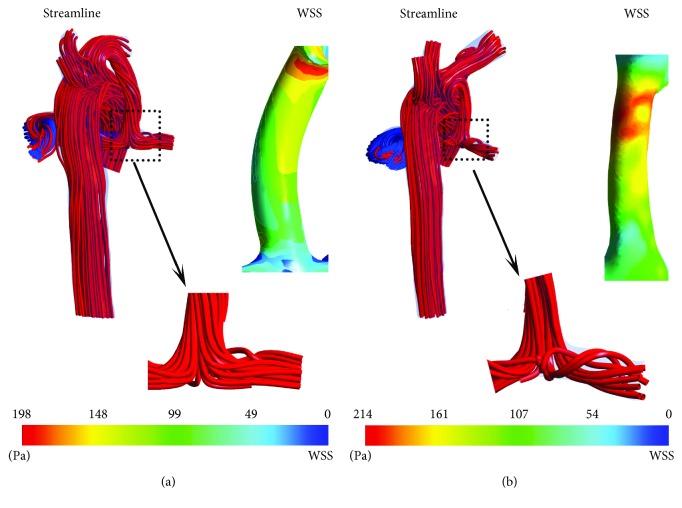
Streamline and wall shear stress (WSS) plots at MBTS before (a) and after (b) surgery for infant B.

There are many factors affecting the development of PA in patients, some of which are hard to predict for specific patients. In this study, the flow state's changes before and after surgery and factors affecting the growth of PA were analysed from the perspective of haemodynamics. The boundary condition of MBTS after surgery was set as the same in Table 2. As is shown in Figure 8, inhomogeneous intraluminal stenosis occurs in the shunt as infant B grows, leading to an increase in shunt resistance and a decrease in shunt ratio; the shunt ratio decreased from 26.14% to 20.55%. Meanwhile, intraluminal stenosis leads to the increase in wall shear stress, which is an important cause of thrombosis. The formation of PA vortices and complex flow structures of PA were highly related to T-junction topology of shunt anastomosis in MBTS, which may result in abnormal PA growth [4]. Comparing the flow states of PA before deformation of RPA, the vorticity of PA is higher when the PRA is bending as is shown in Figure 8. With the growth of patients, on the one hand, the eddy current in PA may increase significantly due to the traction phenomenon caused by insufficient reserved length of the shunt; on the other hand, the inhomogeneous narrowing of the shunt and bending of anastomosis increase flow resistance leading to pulmonary flow and flow of RPA decreasing. For patients with MBTS, the traction phenomenon is unfavourable for symmetric development of LPA and RPA. Therefore, the prediction of PA development based on patients with MBTS should take into account not only the influence of the PA structure on symmetric development of LPA and RPA but also the influence of traction phenomenon when the length of a reserved shunt is insufficient. Although increasing the length of the shunt will lead to an increase in energy loss and a decrease in shunt ratio, a sufficient reserved length of MBTS according to patient's preoperative specific conditions could prevent the occurrence of traction phenomenon and is conducive to symmetrical development of LPA and RPA after operation.

The infants A and B selected in our study showed asymmetrical development of PA after operation. For infant A with CS, asymmetric flow of LPA and RPA was induced by an abnormal PA structure before operation, which resulted in asymmetric development of LPA and RPA after operation. The structure of PA is symmetrical before operation in patients B treated with MBTS, but the traction phenomenon of MBTS has a negative impact on postoperative symmetrical development of PA.

## 4. Discussion

The common problem for the prognosis of SPS is highly related to overflow and underflow. Overflow means excessive shunt ratio and reduction in systemic circulation, which may bring about complications such as congestive heart failure. Underflow indicated that PA flow is insufficient and oxygen saturation in the blood is too low to reach the ideal result of operation. Our results demonstrated that CS has higher PA blood flow rate compared to MBTS. To ensure sufficient PA flow and prevent complications such as congestive heart failure, CS could be preferred for cases with very low PA overflow risk and MBTS for high PA overflow risk. The complex flow structures observed in RPA and LPA may lead to abnormal PA growth. Simulation results shows that velocity distribution in LPA and RPA was relatively uniform in CS, which is consistent with the study by Bao et al. [31]. Nevertheless, an obvious swirling phenomenon occurred at the RPA in MBTS resulting in formation of high vorticity regions.

Whether to retain the MPA depends on the specific condition of the patient. It is notable that the flow state of SPS with APBF is different from the flow state of SPS without APBF. Energy loss of CS is higher than that of MBTS when there is still APBF, while the conclusion is opposite when MPA was transected [4]. For patients with underdeveloped myocardium, energy loss is an important evaluation parameter. Therefore, whether to retain MPA has certain influence on the choice of an optimal operation plan.

With the growth of infants, who are affected by high pressure gradients and varying flow pulsatility, SPS often develops uneven intraluminal narrowing or curvature distortion during the first months after implantation [28]. For infants treated with CS and MBTS, the stenosis or deformation of the shunt after operation will lead to an increase in shunt resistance and a decrease in the shunt ratio. In addition, the increase of maximum shear stress and high shear stress region may lead to thrombosis and a series of complications such as intimal hyperplasia [29, 30] after distortion of the shunt.

Postoperative development of PA is the most common concern of SPS. The development of LPA and RPA for patients treated with CS is closer to natural development, and the probability of LPA and RPA distortion is very small in the long term. However, abnormal preoperative PA structure will lead to asymmetric development of LPA and RPA after surgery like infant A in our study. The LPA/RPA ratio of MBTS approaches unity when compared with CS. Nevertheless, when the length of reserved MBTS shunt is insufficient, traction phenomenon may occur, leading to asymmetrical development of PA as seen in infant B in our study.

Although the lumped parameter method has been widely used and recognized in biomechanics, it still has some deviations due to lack of clinical experiments. In addition, the elasticity of the vascular wall was neglected in this study, and the fluid-solid coupling method will be considered in the next work.

## 5. Conclusion

For specific patients, the selection of shunt configurations should take into account the shunt ratio, energy loss, LPA/RPA split flow ratio, and other parameters. Because of the high shunt ratio, CS could be preferred for patients with very low PA overflow risk.

MBTS could be preferred for cases with underdeveloped myocardium owing to low energy loss. With the growth of infants, the shunt ratio of infants decreases, but maximum shear stress and distribution regions of high shear stress will increase, which raise the probability of thrombosis. Velocity distribution of CS in LPA and RPA is uniform, which is closer to natural development; however, the symmetrical development of LPA and RPA is greatly influenced by the preoperative PA structure. The LPA/RPA ratio of MBTS approaches unity compared with CS, but an insufficient length of reserved MBTS shunt will lead to traction phenomenon and increased eddy current in PA, which is not conducive to symmetrical development of LPA and RPA.

## Figures and Tables

**Table 1:** Values of parameters in LPM.

Block	R (mmHg·s/ml)		C (ml/mmHg)		L (mmHg·s^2^/ml)	
AAQ	R1	0.12	C1	0.127	L1	0.0013
IA	R2	1.69	C2	0.0962	L2	0.00038
LCA	R3	1.858	C3	0.08911	L3	0.00047
LSA	R4	1.858	C4	0.08911	L4	0.00047
RUA	R5	1.065	C5	0.1399	L5	0.00213
LUA	R6	0.5751	C6	0.1463	L6	0.00107
SVC	R7	1.235	C7	0.7233	L7	0.0005
LEA	R8	0.863	C8	0.01995	L8	0.001248
IVC	R9	1.453	C9	0.8571	L9	0.000977
PC	R10	0.0075	C10	0.02394	L10	0.00049
	R11	0.1002	C11	0.00465	L11	0.000488
	R12	0.0075	C12	0.02394	L12	0.00049
	R13	0.1002	C13	0.00465	L13	0.000488
Cardiac circulation	R14	0.015	C14	0.0409		
	R15	0.135	C15	0.009975		
	R16	3.45				
	R17	0.0213				
Shunt	Rcs	3.0075				
	Rmbts	2.6316				
	R18	0.0676				
	R19	0.0676				
	R20	0.0676				
	R21	0.0133				

**Table 2:** Haemodynamic variables of CS computed by lump parameters model.

Boundary condition	AAO (m/s)	MPA (m/s)	LSA (mmHg)	IA (mmHg)	LCA (mmHg)	DAO (mmHg)	LPA (mmHg)	RPA (mmHg)
Mean value	0.6	0.1	78.52	78.66	78.57	78.53	30.85	30.85

**Table 3:** Haemodynamic variables of MBTS computed by lump parameters model.

Boundary condition	AAO (m/s)	MPA (m/s)	LSA (mmHg)	IA (mmHg)	LCA (mmHg)	DAO (mmHg)	LPA (mmHg)	RPA (mmHg)
Mean value	0.6	0.1	77.68	77.51	77.46	77.7	31.32	31.32

## Data Availability

Previously reported data were used to support this study and are available at R726.5; R318.01; R318.01; 10.1152/ajpheart.2001.280.5.H2076. These prior studies (and datasets) are cited at relevant places within the text as references [5, 17, 21].
